# Synergic effect of combined xenogeneic mesenchymal stem cells and ceftriaxone on acute septic arthritis

**DOI:** 10.1093/stcltm/szae034

**Published:** 2024-06-03

**Authors:** Pei-Hsun Sung, Tsung-Cheng Yin, John Y Chiang, Chih-Hung Chen, Chi-Ruei Huang, Mel S Lee, Hon-Kan Yip

**Affiliations:** Division of Cardiology, Department of Internal Medicine, Kaohsiung Chang Gung Memorial Hospital and Chang Gung University College of Medicine, Kaohsiung 833401, Taiwan, ROC; Center for Shockwave Medicine and Tissue Engineering, Kaohsiung Chang Gung Memorial Hospital Kaohsiung, Kaohsiung 833401, Taiwan, ROC; Institute for Translational Research in Biomedicine, Kaohsiung Chang Gung Memorial Hospital Kaohsiung 833401, Taiwan, ROC; Department of Orthopedics, Kaohsiung Chang Gung Memorial Hospital and Chang Gung University College of Medicine, 833401 Kaohsiung, Taiwan, ROC; Center for General Education, Cheng Shiu University, Kaohsiung 833301, Taiwan, ROC; Department of Computer Science and Engineering, National Sun Yat-Sen University, Kaohsiung 804201, Taiwan, ROC; Department of Healthcare Administration and Medical Informatics, Kaohsiung Medical University, Kaohsiung 807378, Taiwan, ROC; Divisions of General Medicine, Kaohsiung Chang Gung Memorial Hospital, Chang Gung University College of Medicine, Kaohsiung 833401, Taiwan, ROC; Division of Cardiology, Department of Internal Medicine, Kaohsiung Chang Gung Memorial Hospital and Chang Gung University College of Medicine, Kaohsiung 833401, Taiwan, ROC; Center for Shockwave Medicine and Tissue Engineering, Kaohsiung Chang Gung Memorial Hospital Kaohsiung, Kaohsiung 833401, Taiwan, ROC; Department of Internal Medicine, Paochien Hospital, Pingtung 900068, Taiwan, ROC; Division of Cardiology, Department of Internal Medicine, Kaohsiung Chang Gung Memorial Hospital and Chang Gung University College of Medicine, Kaohsiung 833401, Taiwan, ROC; Center for Shockwave Medicine and Tissue Engineering, Kaohsiung Chang Gung Memorial Hospital Kaohsiung, Kaohsiung 833401, Taiwan, ROC; Institute for Translational Research in Biomedicine, Kaohsiung Chang Gung Memorial Hospital Kaohsiung 833401, Taiwan, ROC; Department of Nursing, Asia University Taichung 413305, Taiwan, ROC; Department of Medical Research, China Medical University Hospital, China Medical University, Taichung 404333, Taiwan, ROC; School of Medicine, College of Medicine, Chang Gung University, Taoyuan 333323, Taiwan, ROC

**Keywords:** acute sepsis arthritis, inflammation, knee joint, vertebral destruction

## Abstract

**Background:**

This study tested the hypothesis that combined ceftriaxone (Cef) and human umbilical cord-derived mesenchymal stem cells (HUCDMSCs) was better than either therapy for alleviating acute septic arthritis (ASA).

**Methods and results:**

Adult-male C57BL/6 mice were categorized into control group (Clt), group A (ASA only), group B [ASA + Cef (5 mg/kg, IM per day, at days 2 to 16 after ASA induction)], group C [ASA + HUCDMSCs (5 × 10^5^ per mice at days 2, 3, 4 after ASA induction)], and group D (ASA + Cef + HUCDMSCs). Animals were euthanized by day 28. The result demonstrated that the body weight was significantly lower, whereas the ratio of kidney or spleen weight to WB, circulatory WBC count, bacterial colony-formation-unit from circulatory/kidney extraction were significantly higher in group A than in other groups (all *P* < .001). The proinflammatory cytokines (IL-6/TNF-α) of knee joint fluid were lowest in Clt and significantly and progressively reduced from groups A to D, whereas the circulatory levels of these 2 parameters at the time points of days 3/7/28 exhibited an identical pattern as knee joint fluid among the groups (all *P*-value < .0001). The scores of vertebral-bone destructions/inflamed synovium were lowest in Clt, highest in group A, significantly higher in group C than in groups B/D, and significantly higher in group C than in group D (all *P* < .0001).

**Conclusion:**

Combined antibiotics and Cef and HUCDMSCs was superior to just one therapy for suppressing circulatory and tissue levels of inflammation and knee joint destruction in ASA.

## Graphical Abstract

Schematically proposed the underlying mechanism of Cef and HUCDMSCs treatment on attenuating the bacterial-induced organ damage. Cef, ceftriaxone; HUCDMSCs, human umbilical cord-derived mesenchymal stem cells; CT, computerized tomography; WBC, white blood cell; CF, colony formation unit; IL, interleukin; TNF, tumor necrosis factor.

## Introduction

*Staphylococcus aureus* (*S. aureus*) is undoubtedly a leading pathogen of bacteremia which commonly leads to infective endocarditis, metastatic abscess formation, toxic shock syndrome, pneumonia, osteomyelitis, and septic arthritis (SA) (ie, so-called osteoarticular infections, all defined as complex bone and joint infections).^1^ Plentiful data have shown that *S. aureus* is the predominant etiologic agent of osteomyelitis.^2-6^ Currently, intravenous administration of antibiotics is one of the standard therapies for complex bone and joint infections.

The treatment of osteoarticular infections remains a formidable challenge.^7^ The long-term utilization of antibiotics has led to the high frequency of development of multidrug-resistant and methicillin-resistant *S. aureus*.^7^ On the other hand, local antibiotic delivery has been used to increase the antimicrobial concentration at the site of infection and avoid potential systemic side effects.^8^ Another standard method for osteoarticular infections (ie, complex bone and joint infections) is typically managed with surgery and a prolonged therapeutic course of either intravenous or oral antibiotic agent.^5,9,10^ However, even with the development of abovementioned antimicrobial therapies, complex bone and joint infections remain a major cause of morbidity worldwide. Whatever standard therapy is administered, the successful and complete therapy is still quite difficult.^11^

Regrettably, one of the hallmarks of osteomyelitis/SA is the large-amount of inflammation that directly involves in the destructive bone.^12^ Histopathological findings clearly demonstrate that *S. aureus* infection is always accompanied by a recruitment of massive inflammatory and immune cells.^13,14^ Additionally, the proinflammatory cytokines, such as TNF-α, IFN-γ, IL-1, IL-2, and IL-6,^15-25^ have also been identified to participate in the pathogenesis of *S. aureus* infection.

Plentiful data have shown that mesenchymal stem cells (MSCs) have intrinsic capacity to attenuate inflammation^26-30^ and suppress innate/adaptive immunity.^26-30^ In experimental and clinical trials, MSC therapy effectively improved ischemia-related organ dysfunction^26-30^ and clinical outcome for patients with severe immunological disorders^31^ and acute respiratory distress syndrome^32^ mainly through suppressing inflammatory-immune reaction. Intriguingly, growing data have identified that MSCs have antimicrobial properties.^33-37^ Additionally, these MSCs have been reported to directly participate in the innate immune response through the secretion of antimicrobial peptides.^33^ Our previous studies have also shown that MSCs therapy exerted antibacterial activity and offered synergic effect with antibiotics to protect the organs against sepsis syndrome.^30,38-40^ The aforementioned issues raised the hypothesis combined ceftriaxone and HUCDMSCs will be superior to merely one for treatment of acute SA in mouse.

## Materials and methods

### Ethical issues

All animal procedures were approved by the Institute of Animal Care and Use Committee at Kaohsiung Chang Gung Memorial Hospital (Affidavit of Approval of Animal Use Protocol No. 2020110901) and performed in accordance with the Guide for the Care and Use of Laboratory Animals.

Animals were housed in an Association for Assessment and Accreditation of Laboratory Animal Care International (AAALAC)-approved animal facility in our hospital with controlled temperature and light cycles (24 ^o^C and 12/12 light cycle).

### Pilot study for elucidating the suitable dosage of *Staphylococcus aureus*-induced sepsis, animal grouping and the experimental protocols for acute septic arthritis (ASA)

Prior to elucidating the suitable dosage of *Staphylococcus aureus* (*S. aureus*)-induced acute septic arthritis (ASA), the adult male with 12-week old C57BL/6NCrl (ie, standard B6) (*n* = 9), weighing 20-25 g (Charles River Technology), were categorized into groups A1 (ie, 7.0 × 10^6^/200 µL of *S. aureus* were intravenously administered for each animal), A2 (ie, 5.0 × 10^6^/200 µL of *S. aureus* were intravenously administered for each animal), and A3 (ie, 2.5 × 10^6^/200 µL of *S. aureus* were intravenously administered for each animal), respectively. Within 7 days after ASA induction, the mortality rate of A1 was 100% and A2 was 66.7% (ie, 2 of 3 died in A2). On the other hand, the mortality rate of A3 was 33.3% (ie, 1 of 3 died in A3). Additionally, the procedure and protocol were also based on the previous reports^41,42^ with minimal modification. Finally, the purpose of this study was to conduct the ASA setting rather than to investigate the mortality, hence, these animals were inoculated intravenously (ie, from tail vein) *S. aureus* subsp. (ATCC® 19636™) (bought from Food Industry Research and Development Institute, Taiwan) (ie, with a dosage of 2.5 × 10^6^/200 µL of *S. aureus*) was employed to induced ASA animal model.

The B6 mice (*n* = 60) were utilized in the present study for ASA induction. Within 2 days prior to categorizing the animals into different groups, 2 animals died. Accordingly, by day 2 after ASA induction these animals were designated into group A [acute septic arthritis (ASA) only; *n* = 14], group B [ASA + ceftriaxone (5 mg/kg), intramuscular administration (IM) per day, since days 2-16 after ASA induction; *n* = 14], group C [ASA + HUCDMSCs (5 × 10^5^ cells in 200 µL normal saline/per mice; *n* = 15) by intravenous administration at days 2, 3 and 4 after ASA induction] and group D (ASA + ceftriaxone + HUCDMSC; *n* = 15). For comparison, additional 8 animals were utilized and served as a normal-control (Clt) group.

The dosage of ceftriaxone to be utilized in the present study was following our previous report with some modifications.^39^ On the other hand, the dosage of HUCDMSCs to be utilized in the present study was based on our previous reports with some modifications.^43,44^

### Rationale of utilizing the male-gender mice in the present study

The gender-based difference in septic response has been reported in previous studies.^45-47^ To compare the female rodents, male rodents have higher susceptibility to sepsis and poorer outcomes in cardiac and immune response to septic shock.^45-47^ Additionally, the female-sex hormone might also affect the pathophysiological response in ASA. Hence, for effective induction of the ASA, we utilized male animals in the present study.

### Source of the HUCDMSCs

The HUCDMSCs were donated from BIONET CORP as a gift for the purpose of scientific study. The HUCDMSCs are commercialized for clinical trial that has been proved by the Taiwan FDA (ie, TFDA). The passage of the HUCDMSCs used in the present study was 5.

### In vitro study of TSA plate culture to test the impact of Cef, MSC-derived condition medium, and combined Cef and MSC-derived condition medium treatment on inhibiting the growth of the bacteria (referred to Supplementary Figures 1, 2, and 3)

In this *in vitro* study, the *Staphylococcus aureus* was cultured overnight in TSB broth and quantified using DEN-1B, McFarland densitometer. Take 1 × 10^8^  *Staphylococcus aureus* in a culture tube, add 2.5 mL of fresh collected MSC-derived condition medium (the control group is replaced by fresh culture medium) or stepwise increased in Cef concentration (ie, 3, 6, and 10 µM), and performed shaking culture at 35 °C. Samples were harvested at the designed time points for TSA plate culture, and colonies were counted after overnight culture.

### Blood samplings for serial changes of circulating level of white blood cell (WBC) count and proinflammatory cytokines and collection of knee joint fluid for evaluation of the proinflammatory cytokines by day 28

The blood samplings were collected at the time points of baseline and days 3, 7, and 28 after ASA induction for measuring the circulatory proinflammatory cytokines, including tumor necrosis factor (TNF)-α (#RTA00, R&D) and interleukin (IL)-6 (#R6000B, R&D) by using the ELISA standard method according to the manufactory recommendation.

Additionally, by the end of the study period (ie, by day 28 after ASA), we first harvested the knee joint, followed by carefully eradicating the muscle, and tendon and removing the distal femoral and proximal tubular bones. The knee joint was then put into the micro-centrifugation tube for centrifugation. Finally, the fluid was collected for the ELISA study for these inflammatory parameters.

### Assessment of time courses of body weight, and ratios of kidney weight and spleen weight to body weight at day 28 just after euthanasia of the animals in each group

To elucidate whether the ASA would affect the growth in the animals, the body weight of each animal was measured at base line and by days 3, 7, 14, 21, and 28 after ASA induction. Additionally, the kidney and spleen weights were also recorded by day 28 after the animals to be euthanized.

### Assessment of bacterial colony-forming unit (CFU) from kidney and blood at day 28 just after harvesting these organs and collecting blood from the animals in each group

To assess whether the bacteria was still present or absent in the circulation and vulnerable organs, the circulatory blood was collected, and kidney tissue was extracted. In detail, 100 µL of blood was drawn from tail vein and diluted with PBS to 500 µL. Additionally, dilution of 100 µL into 10 × (ie, second dilution) and 100 × (ie, third dilution) was conducted, respectively. These dilutions were scribbled (ie, smearing) on the agar plate and incubated for 16 hours. Finally, the CFUs were counted.

Similarly, the kidney tissue was extracted and homogenously minced and finally immersed in 500 µL PBS. The dilution procedure followed and proceeded exactly as that of circulatory blood mentioned in the previous paragraph.

### Methodology of decalcification of bone and joints for assessment of ASA-induced articular and cartilage destruction

To well prepare for microscopic findings of H&E staining for identification of ASA-induced articular and cartilage destruction as well as the histopathologic feature of inflamed synovium, decalcification was conducted. In detail, after harvesting the specimens, the attached soft tissues on the bone-articular joints were carefully eradicated. These bone-articular joints (knee joints, distal femoral, and proximal tubular bone) were then immersed into the on centrifugation tube with 50 mL BPS and 10 mL of Decalcifier II solution (Leica, CAS No: 7647-01-0) for 3 days. Afterward, the needle was utilized to carefully puncture the specimen. The completion of decalcification corresponded to the void of resistance. After decalcification, the specimens were then immersed in 2% NaOH for 1-2 hours, followed by washing with water for 30 minutes; finally, paraffin embedding for assessment of bone/cartilage destruction was conducted.

### Utilization of micro-CT to measure the bone, joint, cartilage erosion, and destructive morphological features by day 28 after ASA induction

By day 28, ie, at the end of the study period, the animals were euthanized, and the bone-articular joints were harvested from each animal. After well preparation, these specimens were assessed by the Bruker Micro-computerized tomography (CT) (SkyScan 1176, Bruker BioSpin, Germany with Bruker CTAn Micro-CT Software for analysis) for elucidating the destructive features of the bone, that is, articular knee joint.

### Immunofluorescent study

Immunofluorescent staining was performed for the examinations of MMP9 (1:200, Invitrogen) and CD68 (1:500, Abcam). Respective primary antibody was used with irrelevant antibodies as controls. Three sections of kidney and spleen specimens were analyzed in each mouse. For quantification, 3 randomly selected high-power fields (HPFs) of the microscope (400× for IF studies) were analyzed in each section. The mean number per HPF for each animal was determined by summation of all numbers divided by 9 (ie, 3 sections of kidney or spleen specimens × 3 randomly selected HPFs = 9. Thus, to get the mean number per HPF for each animal had to divided by 9). Additionally, the tissue slides were interpreted by an expert of rodent pathology.

### Western blot analysis of left kidney specimens

Equal amounts (50 μg) of protein extracts were loaded and separated by SDS–PAGE using acrylamide gradients. After electrophoresis, the separated proteins were transferred electrophoretically to a polyvinylidene difluoride (PVDF) membrane (Amersham Biosciences). Nonspecific sites were blocked by incubation of the membrane in a blocking buffer [5% nonfat dry milk in T-TBS (TBS containing 0.05% Tween 20)] overnight. The membranes were incubated with the indicated primary antibodies [matrix metalloproteinase (MMP9) (1:1000, Abcam), interleukin (IL)-1β (1:1000, Cell Signaling), and tumor necrosis factor (TNF)-α (1:1000, Cell Signaling) and actin (1: 10 000, Millipore)] for 1 hour at room temperature. Horseradish peroxidase-conjugated anti-rabbit immunoglobulin IgG (1:6000, Sigma) and anti-mouse immunoglobulin IgG (1:6000, Sigma) were used as a secondary antibody for 1-hour incubation at room temperature. The washing procedure was repeated to 8 times within 1 hour. Immunoreactive bands were visualized by enhanced chemiluminescence (ECL; Amersham Biosciences) and exposed to Biomax L film (Kodak). For the purpose of quantification, ECL signals were digitized using Labwork software (UVP).

Note that in this study, we applied 6 animals per group for each experiment, indicating 6 sets of samples were prepared for each western blotting. In each set of samples, we utilized the antibody against β-actin for loading control and normalization. Therefore, if the presented pictures of western blotting against different proteins were derived from the same set of samples, the image of actin for normalization should be the same one.

### Scoring for synovial hyperplasia and destruction of bone-articular knee joint

The scoring of hyperplasia of synovium (ie, synoviocyte hypertrophy) of the knee joint was defined as 0 = healthy synovium; 1 = mild hyperplasia; 2 = moderate hyperplasia; and 3 = severe hyperplasia. The scoring was based on the previous report^48^ with some modifications.

The scoring of bone-articular knee joint destruction was defined as 0 = healthy joint; 1 = mild bone destruction; 2 = moderate bone destruction; and 3 = severe bone destruction. The scoring was based on the previous report^44^ with some modifications.

### Statistical analysis

Quantitative data were expressed as mean ± SD. Statistical analysis was adequately performed by ANOVA, followed by Bonferroni multiple comparison post hoc test. SAS statistical software for Windows version 8.2 (SAS Institute) was utilized. A *P* value < .05 was considered statistically significant.

## Results

### To evaluate the mortality rate, serial changes of body weight, and ratio of kidney and splenic weight to body weight at day 28

**Figure 1.**
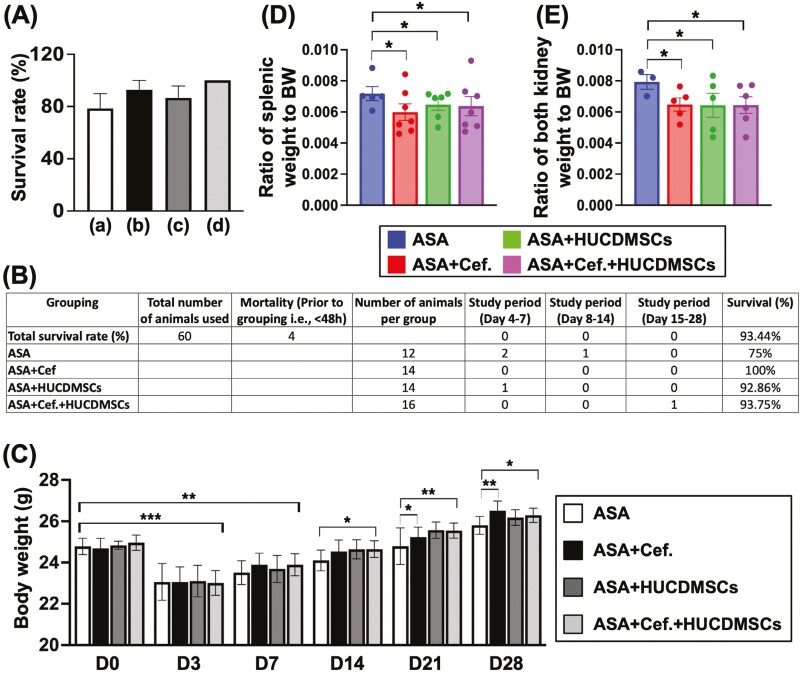
Accumulated mortality, serial changes of body weight, and body weight of the kidneys and spleen by day 28 after ASA induction. (A) Accumulated mortality rate in groups (a) 21.4% (3/14), (b) 7.1% (1/14), (b) 13.3% (2/15), and (d) 0% (0/15), *P* = .27 (by Chi-square test). (B) Illustrating the number of the animals to be utilized, number of mortalities prior to grouping, number of animals among the groups just after grouping and the survival rate at the end of the study period. (C) At baseline, and days 3 and 7, the body weight (BW) did not differ among the groups. However, as compared to the baseline the BW was notably reduced at days 3 and 7, ** indicated *P* value < .01; *** indicated *P* value < .001. By days 14, 21, and 28 after ASA induction, the BW was significantly reduced in ASA group (white bar) than in other groups, * indicated *P* < .05; ** indicated *P* value < .01. (D) Ratio of splenic weight to BW by day 28 was significantly lower in ASA group than in other groups, * indicated *P* < .05. (E) Ratio of both kidney weight to BW by day 28 was significantly lower in ASA group than in other groups, * indicated *P* < .05. Abbreviations: ASA, acute septic arthritis; Cef, ceftriaxone; HUCDMSCs, human umbilical cord-derived mesenchymal stem cells.

The mortality rate at day 2 after ASA induction prior to grouping was 3.3% (ie, 2 of 60 animals) (Figure 1B). On the other hand, by the end of study period, the mortality rate in groups A (ASA), B (ASA + ceftriaxone), C (ASA + HUCDMSCs), D (ASA + ceftriaxone + HUCDMSC), and the Clt was 21.4% (3/14), 7.1% (1/14), 13.3% (2/15), 0% (0/15), and 0% (0/8), respectively (Figure 1B). Thus, the mortality rate did not differ among the groups (*P* = 0.27 by Chi-Square test) (Figure 1A and 1B).

As compared to the baseline (ie, at day 0 just before ASA induction), the body weight in each group (ie, comparison among A, B, C, and D) was significantly reduced at days 3 and 7 after ASA induction that was notably progressively increased after days 7-28 after ASA induction (Figure 1C). Additionally, the body weight was notably lower in group A than in other groups at the time points of days 7, 14, 21, and 28 after ASA induction (Figure 1C).

On the other hand, the ratio of both splenic weight (Figure 1D) and kidney weight (Figure 1E) to the body weight at day 28 after ASA induction was significantly higher in group A than in other groups but it did not differ among the B, C, and D, suggesting that an increase in ratio of kidney weight and splenic weight to body weight could mainly be because the body weight was notably lower in A after sepsis induction.

### Synergic effect of combined ceftriaxone and HUCDMSC on suppressing circulating and knee articular fluid levels of the proinflammatory cytokines

**Figure 2.**
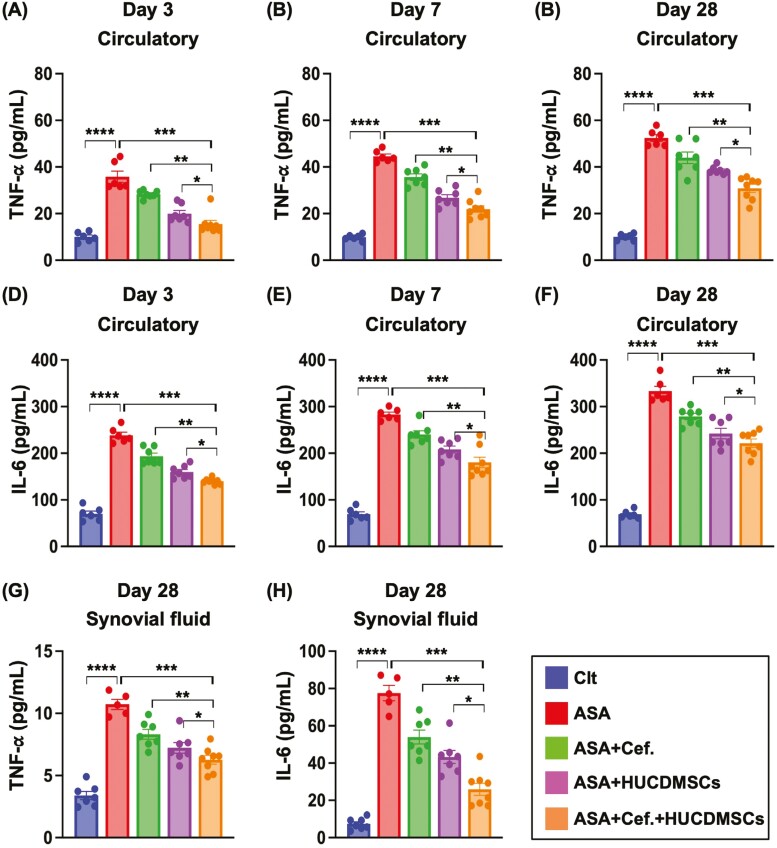
Time courses of circulating levels of the proinflammatory cytokines and these inflammatory cytokines at the time point of day 28 in knee articular fluid after ASA induction. (A) Serum level of tumor necrosis factor (TNF)-α by day 3, * indicated *P* < .05; ** indicated *P* < .01; *** indicated *P* < .001; **** indicated *P* < .0001. (B) Serum level of TNF-α by day 7, * indicated *P* < .05; ** indicated *P* < .01; *** indicated *P* < .001; **** indicated *P* < .0001. (C) Serum level of TNF-α by day 28, * indicated *P* < .05; ** indicated *P* < .01; *** indicated *P* < .001; **** indicated *P* < .0001. (D) Serum level of interleukin (IL)-6 by day 3, * indicated *P* < .05; ** indicated *P* < .01; *** indicated *P* < .001; **** indicated *P* < .0001. (E) Serum level of IL-6 by day 7, * indicated *P* < .05; ** indicated *P* < .01; *** indicated *P* < .001; **** indicated *P* < .0001. (F) Serum level of IL-6 by day 28, * indicated *P* < .05; ** indicated *P* < .01; *** indicated *P* < .001; **** indicated *P* < .0001. (G) Knee articular fluid (ie, normal saline washed synovial fluid) level of TNF-α at day 28, * indicated *P* < .05; ** indicated *P* < .01; *** indicated *P* < .001; **** indicated *P* < .0001. (H) Knee articular fluid (ie, normal saline washed synovial fluid) level of IL-6 at day 28, * indicated *P* < .05; ** indicated *P* < .01; *** indicated *P* < .001; **** indicated *P* < .0001. *n* = 7 for each group. Abbreviations: ASA, acute septic arthritis; Cef, ceftriaxone; Clt, normal control; HUCDMSCs, human umbilical cord-derived mesenchymal stem cells.

To evaluate the status of systemic and local inflammatory reaction, we drew the blood samplings at the time points of days 3, 7, and 28 and the knee articular fluid at day 28 before the euthanasia of the animals. The result demonstrated that the circulatory levels of TNF-α (Figure 2A-2C) and IL-6 (Figure 2D-2F), 2 indicators of inflammation, were lowest in the normal control (Clt) and significantly progressively reduced from A to D regardless of the time points at days 3, 7, or 28. Additionally, these 2 parameters of knee articular fluid (ie, synovial fluid) at day 28 also expressed an identical pattern of circulatory level among the groups (Figure 2G and 2H). Our findings implicated that combined antibiotics and MSCs was superior to merely one for attenuating the inflammatory response in an ASA setting.

### Combined 2 regimens were superior to merely one in attenuating the synovial hyperplasia and bone destruction of the knee joint

**Figure 3.**
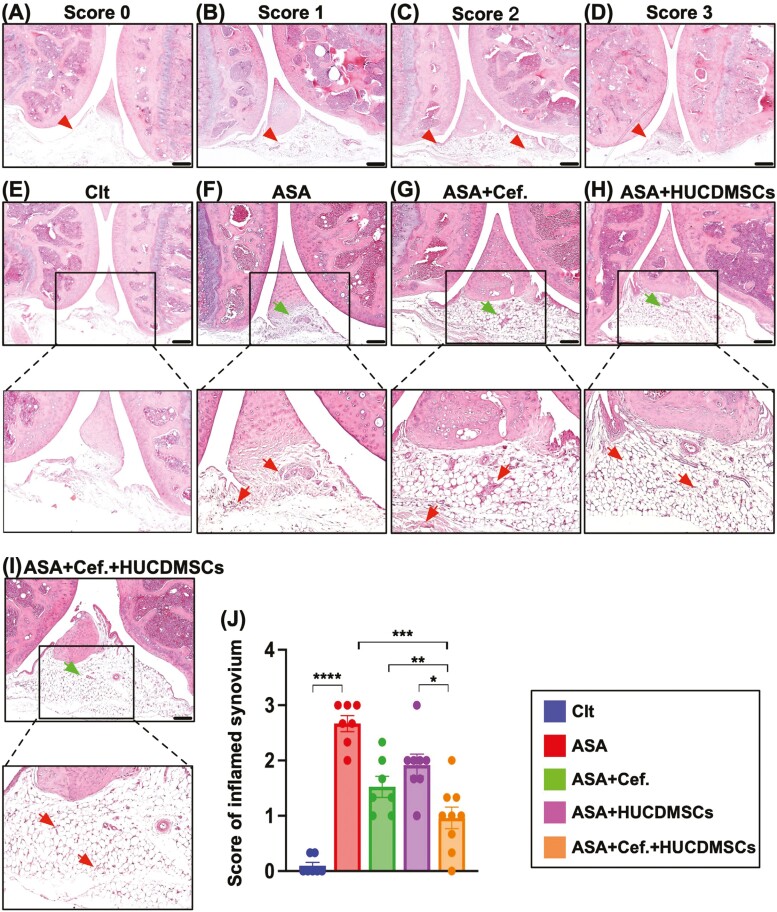
Light microscopic finding for identification of synovial hyperplasia of arthritic knee. (A-D) Illustrating the light microscopic finding (100×) of H&E stain for classification of the scoring of synovial hyperplasia from score 0 to score 3. Scoring of synovial hyperplasia was based on previous report^44^ with some modification: score 0 = synovium without hyperplasia; score 1 = mild synovial hyperplasia; score 2 = moderate synovial hyperplasia; score 3 = severe synovial hyperplasia (red arrow heads). (E-I) Illustrating the light microscopic finding (100×) of H&E stain for identification of hyperplasia of synovium (green arrows) among the groups. Square box indicated the manifestation of synovial area for identification of synoviocyte hyperplasia (red arrows). (J) Analytical result of synovial hyperplasia scoring, * indicated *P* < .05; ** indicated *P* < .01; *** indicated *P* < .001; **** indicated *P* < .0001. Scale bar in right lower corner represents 100 µm. *n* = 8 for each group. Abbreviations: ASA, acute septic arthritis; Cef, ceftriaxone; Clt, normal control; HUCDMSCs, human umbilical cord-derived mesenchymal stem cells.

**Figure 4.**
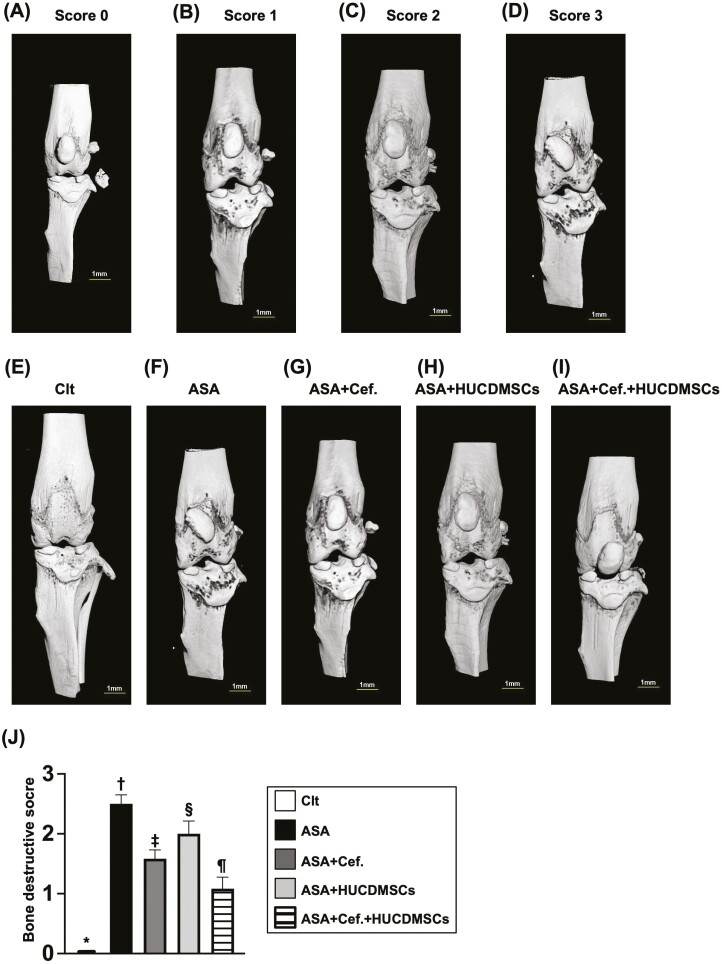
Micro-CT finding for identification of bone destruction on articular knee joint. (A-D) Showing the micro-CT finding for classification of bone destruction (ie, articular knee joint) from scores 0 to 3. Definition of bone destruction scoring (ie, presenting as punctual lesions) by 3-D micro-CT was based on previous report^48^: score 0 = an intact of the knee joint; score 1 = the knee joint of mild bone destruction on the proximal tibia; score 2 = a knee joint with moderate bone erosion (ie, numerous punctual lesions on the proximal tibia and the distal femur (green arrows); score 3 = a heavily destructed knee joint on the distal femur with abundant punctual lesions (pink arrows). (E to I) Illustrating the 3-D micro-CT finding for identification of the bone destructive lesions in knee joints among the groups. (J) Analytical result of bone destructive score, * indicated *P* < .05; ** indicated *P* < .01; *** indicated *P* < .001; **** indicated *P* < .0001. Scale bar in right lower corner represents 1 mm. *n* = 8 for each group. Abbreviations: ASA, acute septic arthritis; Cef, ceftriaxone; Clt, normal control; HUCDMSCs, human umbilical cord-derived mesenchymal stem cells Clt, normal control.

To elucidate the impact of *S. aureus* on the synovial hyperplasia of the arthritic knee, the H&E staining for the decalcified articular knee joint was employed in the present study. Additionally, for identification of the destructions of the knee joint, we employed the micro-CT instrument in the present study. The result of the H&E staining demonstrated that the hyperplasia of synovium was lowest in Clt, highest in A, significantly higher in C than in B and D, and significantly higher in B than in D (Figure 3). Additionally, the destructive score of arthritic knee joint displayed a similar pattern of synovial hyperplasia among the groups. These findings implicated that the combined regimen was better than just one therapy on safeguarding the integrity of the articular knee joint in ASA setting (Figure 4).

### Combined ceftriaxone and HUCDMSC treatment effectively suppressed the WBC count in circulation and CFU in circulation and kidney

**Figure 5.**
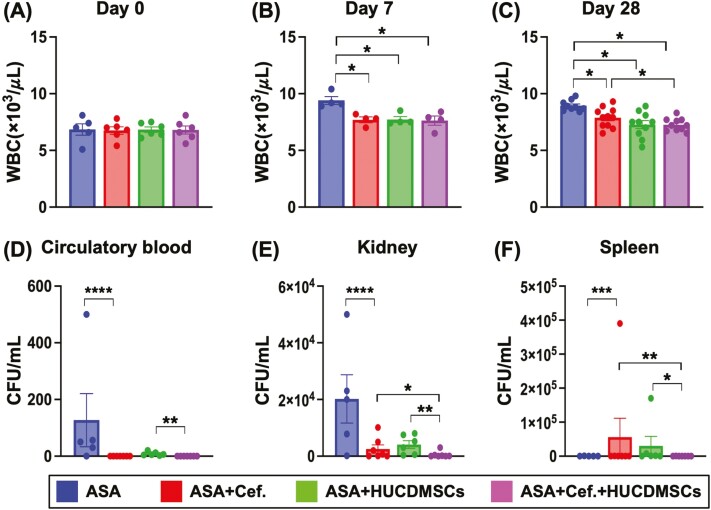
Serial changes of WBC count in circulation and CFU in circulation and kidney by day 28 after ASA induction. (A) Circulating level of white blood cell (WBC) count at baseline, *P* > 0.5. (B) Circulating level of WBC count by days 7, * indicated *P* < .05. (C) Circulating level of WBC count by days 28, * indicated *P* < .05. (D) Colony formation unit (CFU) (ie, bacterial count) from circulatory blood sampling, ** indicated *P* < .01; **** indicated *P* < .0001. (E) The number of CFU in kidney extraction by day 28, * indicated *P* < .05; ** indicated *P* < .01; **** indicated *P* < .0001. (F) The number of CFU in splenic extraction by day 28, * indicated *P* < .05; ** indicated *P* < .01; *** indicated *P* < .0001. *n* = 5-7 for each group. Abbreviations: ASA, acute septic arthritis; Cef, ceftriaxone HUCDMSCs, human umbilical cord-derived mesenchymal stem cells.

It is well-known that measurement of the WBC count is a simple method for identifying not only a situation of inflammation but also the severity of inflammatory response. As we expected, the baseline level of WBC count did not differ among the 4 groups (ie, A-D) (Figure 5A). However, as compared with baseline level, this parameter was significantly increased by days 7 (Figure 5B) and 28 (Figure 5C) after ASA induction among the 4 groups. Additionally, this parameter was significantly higher in group A than in B-D, but it showed no difference among B-D at the time point of day 7 (Figure 5C). Furthermore, at the time point of day 28 after ASA induction this parameter was significantly increased in A than in B-D and significantly increased in B than in C and D, but it showed similarly between C and D (Figure 5C).

The CFU (ie, bacterial count) from circulatory blood sampling was significantly higher in A than in B to D and significantly higher in C than in B and D, but it showed no difference between the latter 2 groups (Figure 5D). Additionally, the mean of CFU in kidney and splenic extractions by day 28 was highest in A, lowest in D, and significantly higher in C than in B (Figure 5E).

### Combined ceftriaxone and HUCDMSC treatment remarkably ameliorated the protein and cellular expressions of inflammatory biomarkers in spleen and kidney

**Figure 6.**
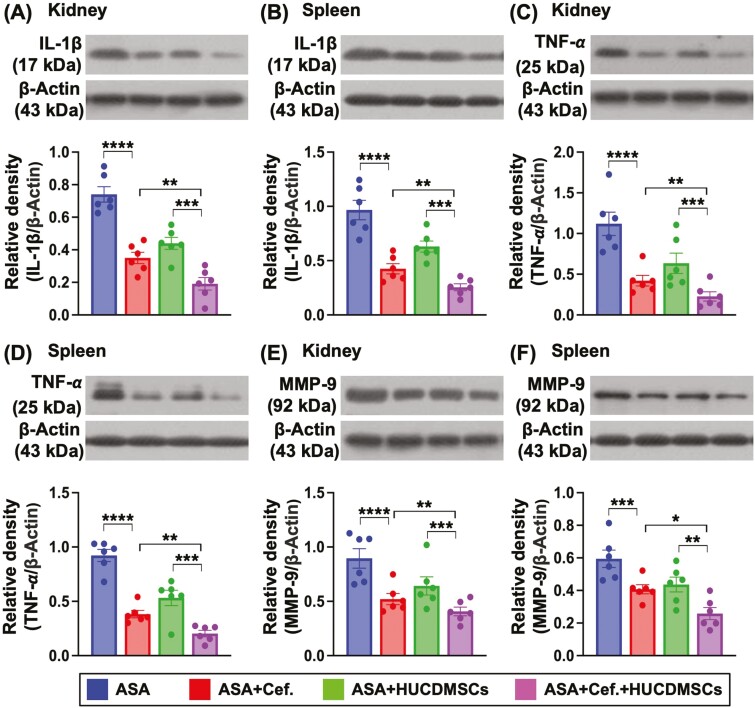
Protein expressions of inflammatory biomarkers in kidney and spleen by day 28 after ASA induction. (A) Protein expression of interleukin (IL)-1β in kidney, * indicated *P* < .05; ** indicated *P* < .01; *** indicated *P* < .001; **** indicated *P* < .0001. (B) Protein expression of IL-1β in spleen, * indicated *P* < .05; ** indicated *P* < .01; *** indicated *P* < .001; **** indicated *P* < .0001. (C) Protein expression of tumor necrosis factor (TNF)-α in kidney, * indicated *P* < .05; ** indicated *P* < .01; *** indicated *P* < .001; **** indicated *P* < .0001. (D) Protein expression of TNF-α in spleen, * indicated *P* < .05; ** indicated *P* < .01; *** indicated *P* < .001; **** indicated *P* < .0001. (E) Protein expression of matrix metalloproteinase (MMP)-9 in kidney, * indicated *P* < .05; ** indicated *P* < .01; **** indicated *P* < .0001. (F) Protein expression of MMP-9 in spleen, * indicated *P* < .05; ** indicated *P* < .01; *** indicated *P* < .001. *n* = 6 for each group. Abbreviations: ASA, acute septic arthritis; Cef, ceftriaxone; HUCDMSCs, human umbilical cord-derived mesenchymal stem cells.

**Figure 7.**
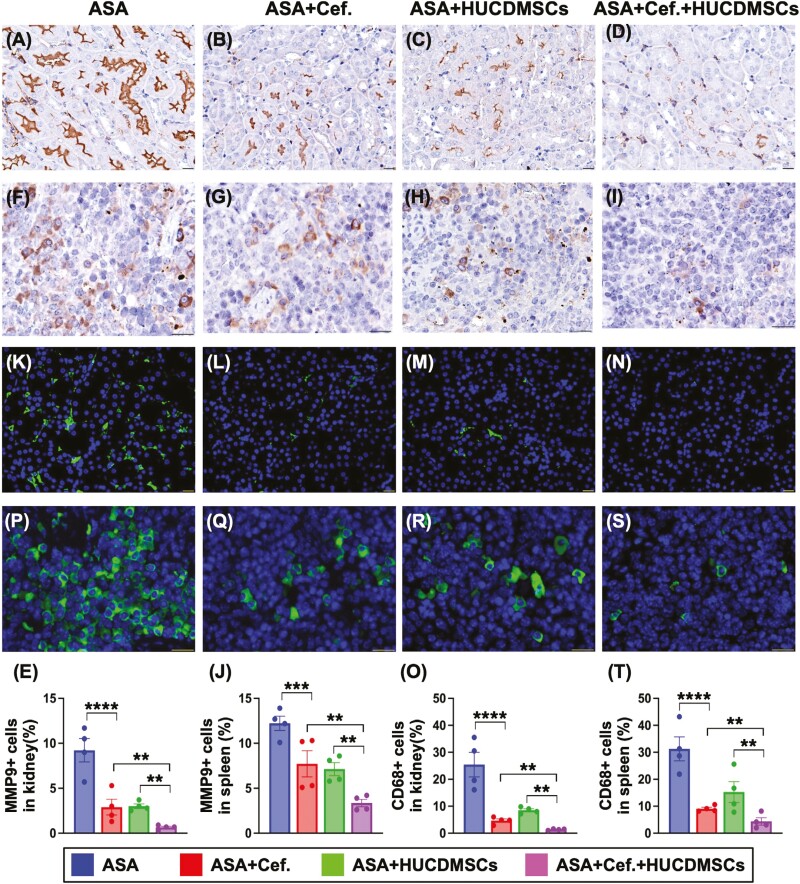
Cellular expressions of Inflammatory biomarkers in kidney and spleen by day 28 after ASA induction. (A-D) Illustrating the immunohistochemical (IHC) microscopic finding (400×) for identification of cellular expression of MMP-9 in kidney. (E) Analytical result of positively stained MMP-9 cells, ** indicated *P* < .01; **** indicated *P* < .0001. (F-I) Illustrating the IHC microscopic finding (400×) for identification of cellular expression of MMP-9 (brown color) in spleen. (J) Analytical result of positively stained MMP-9 cells, ** indicated *P* < .01; *** indicated *P* < .0001. (K-N) Illustrating the immunofluorescent (IF) microscopic finding (400×) for identification of cellular expression of CD68 (green color) in kidney. (O) Analytical result of positively stained CD68 cells, * indicated *P* < .05; ** indicated *P* < .01; **** indicated *P* < .0001. (P-S) Illustrating the IF microscopic finding (400×) for identification of cellular expression of CD68 (green color) in spleen. (T) Analytical result of positively stained CD68 cells, * indicated *P* < .05; ** indicated *P* < .01; **** indicated *P* < .0001. Scale bar in right lower corner represents 20 µm. *n* = 6 for each group. ASA, acute septic arthritis; Cef, ceftriaxone; HUCDMSCs, human umbilical cord-derived mesenchymal stem cells.

To investigate the molecular levels of inflammatory biomarkers presented in spleen and kidney organs, the specimens of these organs were harvested for western analysis. As we expected, the protein expressions of IL-1β (Figure 6A and 6B), TNF-α (Figure 6C and 6D), and MMP-9 (Figure 6E and 6F), 3 indices of inflammation, were significantly higher in A than in B-D, significantly higher in C than in B and D and significantly higher in B than in D. Additionally, when we examined the cellular level of the inflammation in these 2 organs, the IF microscopic finding was utilized in the present study. The result demonstrated that the cellular expressions of MMP-9 (Figure 7A-7I) and CD68 (Figure 7K-7S), 2 indicators of inflammation,^49^ exhibited an identical manner of protein levels of inflammation in Figure 6 (Figure 7E, 7J, 7O, 7T), suggesting that MSCs-facilitated antibiotic was an innovative therapy for ASA.

### *In vitro* study tested the impact of MSC-derived condition medium, Ceftriaxone, and combined these 2 regimens on suppressing the growth of *S. aureus*

To elucidate the therapeutic impact of **Ceftriaxone**, an MSC-derived condition medium and combine these two therapeutic strategies in inhibiting the growth of *S. aureus*, the *in vitro* study was conducted in the present study. As expected, the bacterial growth was significantly suppressed at low concentration (3.0 µM), further significantly suppressed at middle concentration (6.0 µM), and furthermore significantly suppressed by higher concentration (10.0 µM) of Cef at the time points of 4-24 h (Supplementary Figure 1). Additionally, the bacterial growth was also significantly inhibited by MSC-derived condition medium (Supplementary Figure 2). Of importance was that combined Cef and MSC-derived condition medium was superior to merely one on inhibiting the bacterial growth (Supplementary Figure 3).

## Discussion

This study which investigated the therapeutic impact of combined antibiotic (ie, ceftriaxone) and xenogeneic MSCs (ie, HUCDMSCs) on ASA rodents begot several preclinical striking implications. First, we successfully created a useful animal model of ASA for the present study and that could be extrapolated in future preclinical studies for testing the therapeutic potential of any kind of MSCs on the treatment of acute or chronic septic arthritis or even the bacterial osteomyelitis. Second, multiple parameters, including circulatory levels and specific organ tissues of inflammation, micro-CT analysis, histopathological examination, and CFU assessment, were collected for more precise diagnosis and predictive of the outcome in the setting of ASA. Third, the result of the present study demonstrated that combined ceftriaxone and HUCDMSCs were superior to merely one therapy for protecting the articular knee joint structures and cartilage against bacterial septic damage, highlighting that MSCs-facilitated antibiotics could offer a great potential for the treatment of ASA, especially to those ASA patients who are refractory to conventional treatment.

Plentiful data have clearly identified that the *S. aureus* infection is always associated with recruitment of massive inflammatory and immune cells^13,14^ and proinflammatory cytokines in the bone marrow, articular joints, and cartilages.^15-25^ One important finding in the present study was that as compared with normal control, the proinflammatory cytokines were significantly increased not only in the circulation but also in the knee articular joint and in the innocent organs (ie, kidney and spleen were investigated in the current study). However, these proinflammatory cytokines in both circulation and the knee-joint fluid as well as in kidney and spleen organs were markedly suppressed by HUCDMSCs treatment, more suppressed by ceftriaxone treatment, and furthermore suppressed by combining these 2 regimens. In this way, our findings, in addition to supporting the findings of previous,^12^ implied that combined antibiotics and MSCs would be better than a single regimen for treatment of ASA setting.

Abundant evidence has established that ASA and osteomyelitis harbored a huge sum of inflammation associated with a circulatory innate immune response that almost always directly participated in the articular cartilage and bone destruction.^12^ Another important finding in the present study was that when we looked at the micro-CT examination, we identified that the destructive score of the articular knee joint was remarkably increased in ASA animals than that of the normal control (ie, Clt) animals. Additionally, the H&E staining further identified that the synovial hyperplasia of the knee joint was notably increased in that of ASA animals than in that of Clt animals. Our findings were comparable with the findings of previous studies.^12^ Of important finding was that ceftriaxone or HUCDMSCs therapy notably reduced and combined ceftriaxone and HUCDMSCs therapy more notably reduced the above-mentioned pathological perturbations in the joint space, cartilage, and bone, once more highlighting that this combined regimen could pose as a potential treatment of clinical settings of ASA, chronic septic arthritis and even for chronic osteomyelitis.

An interesting finding was that when looking at the number of CFU, a unit that estimates the number of microbial cells, we found that this parameter in kidney, spleen, and circulation was notably increased in A (ie, ASA only) and C (ie, ASA + HUCDMSCs) than in B (ASA + ceftriaxone) and D (ASA + ceftriaxone + HUCDMSCs) but it did not differ between the former or the latter 2 groups. Our findings emerged 2 fundamental implications. First, MSCs therapy could not kill the local or systemic bacteria. Surprisingly, when looking at our animal model studies^38,39^ and clinical trial,^32^ we found that MSCs treatment reduced the mortality and improved the outcomes of sepsis syndrome^38,39^ and acute respiratory distress syndrome caused by sepsis.^32^ Our present and previous^32,38,39^ findings supported that MSCs therapy improved the prognostic outcomes through anti-inflammation and immunomodulation rather than by directly eradicating the bacteria. Second, in sepsis syndrome, bacteria persistently involved in numerous major vital organs could be a common phenomenon, implying that a short interval of MSCs therapy or even a two-week duration of antibiotics in the present was not enough. These raise the need for consideration for the optimization of the dosage and therapeutic duration of ceftriaxone and HUCDMSCs for the rodents as well as the extension of this consideration to our clinical practice in the near future for ASA setting.

### Study limitation

This study has limitations. First, this study was designed to test the impact of ceftriaxone-HUCDMSCs on protecting the bone-articular joint and cartilage against the ASA damage rather than to estimate the impact of these 2 regimens on reducing the mortality rate. Second, without testing the stepwise increased dosage of ceftriaxone or HUCDMSCs on protecting the joint, bone, and other major organs in ASA rodents, we did not know whether the ceftriaxone treatment was superior to HUCDMSCs or vice versa in this preclinical-setting of ASA. Third, in consideration of MSCs being trapped in the lung organ, we could not completely rule out that the effects of HUCDMSCs treatment on protecting the bone-articular-cartilage organ against ASA damage could be, at least in part, due to the effect of exosomes/condition medium which were secreted by HUCDMSCs.

**Figure 8.**
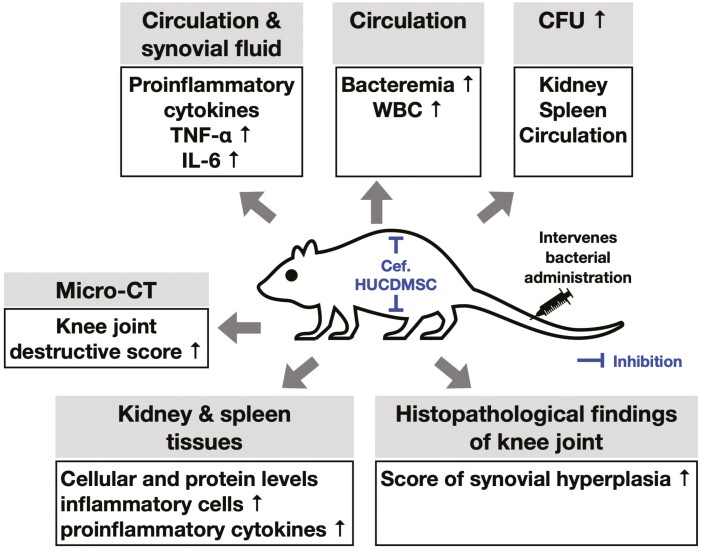
**Schematically proposed the underlying mechanism of Cef and HUCDMSCs treatment on attenuating the bacterial-induced organ damage**.

Although extensive works were done in the present study, the exact underlying mechanism of Cef and HUCDMSCs treatment for ameliorating the ASA-induced organ damage remains uncertain and could be schematically proposed in Figure 8, which was based on our experimental findings.

In conclusion, the results of our study demonstrated that HUCDMSCs-facilitated ceftriaxone therapy protects the bone-articular-cartilage organ against ASA damage. Our findings may encourage the utilization of these combined regimens to treat patients who are refractory to traditional standardized therapy.

## Supplementary material

Supplementary material is available at *Stem Cells Translational Medicine* online.

szae034_suppl_Supplementary_Material

## Data Availability

The data that support the findings of this study are available from the corresponding authors upon reasonable request.
