# Supplementary material for: Synergic effect of combined xenogeneic mesenchymal stem cells and ceftriaxone on acute septic arthritis
Source: Stem Cells Transl Med. 2024 Jun 3;13(8):724–37. doi: 10.1093/stcltm/szae034 (PMC11328939; doi:10.1093/stcltm/szae034)
Supplement: szae034_suppl_Supplementary_Material [file szae034_suppl_supplementary_material.zip › Supplementary Figure/Supplementary Figure.docx]

**Supplementary Figure 1. Impact of Cef therapy on suppressing the *Staphylococcus aureus* growth**

**Left panel)** Illustrating the bacterial growth at the time points of 4 and 8 h in culture plate. The bacterial growth was remarkably and further remarkably suppressed as the Cef concentration progressively increased from 3µM to 10µM. **Right panel)** Illustrating the time points (i.e., 0, 2, 4, 6, 8, 24 h) of colony formation unit (CFU) in culture plate. As compared to the control group, the CFU was notably suppressed by Cef in these time points. Additionally, this parameter was remarkably and more progressively suppressed as the concentration of Cef increased (n = 3 for each time points). Cef = ceftriaxone.

**Supplementary Figure 2.** **Impact of mesenchymal stem cells (MSCs)-derived condition medium therapy on suppressing the *Staphylococcus aureus* growth**

**Left panel)** Showing the morphological feature of bacterial growth at the time points of 4 and 8 h in culture plate. The bacterial growth was markedly suppressed by MSCs-derived condition medium treatment. **Right panel)** Demonstrating the time points (i.e., 0, 2, 4, 6, 8, 24 h) of colony formation unit (CFU) in culture plate. As compared with control group, the CFU was notably suppressed in these time points (n = 3 for each time points).

**Supplementary Figure 3.** **Impact of combined of MSCs-derived condition medium therapy on suppressing the *Staphylococcus aureus* growth**

**Left panel)** Showing the morphological feature of bacterial growth at the time points of 4 and 8 h in culture plate. The bacterial growth was remarkably suppressed by MSCs-derived condition medium treatment and more remarkably suppressed by combined Cef and MSCs-derived condition medium treatment. **Right panel)** Showing the time points (i.e., 0, 2, 4, 6, 8, 24 h) of colony formation unit (CFU) in culture plate. As compared with control group, the CFU was remarkably suppressed by MSCs-derived condition medium and more remarkably suppressed by the two regimens in these time points of 0, 2, 4, 6, 8 and 24 h (n = 3 for each time points).
