# Supplementary figures and images for: Synergic effect of combined xenogeneic mesenchymal stem cells and ceftriaxone on acute septic arthritis
Source: Stem Cells Transl Med. 2024 Jun 3;13(8):724–37. doi: 10.1093/stcltm/szae034 (PMC11328939; doi:10.1093/stcltm/szae034)

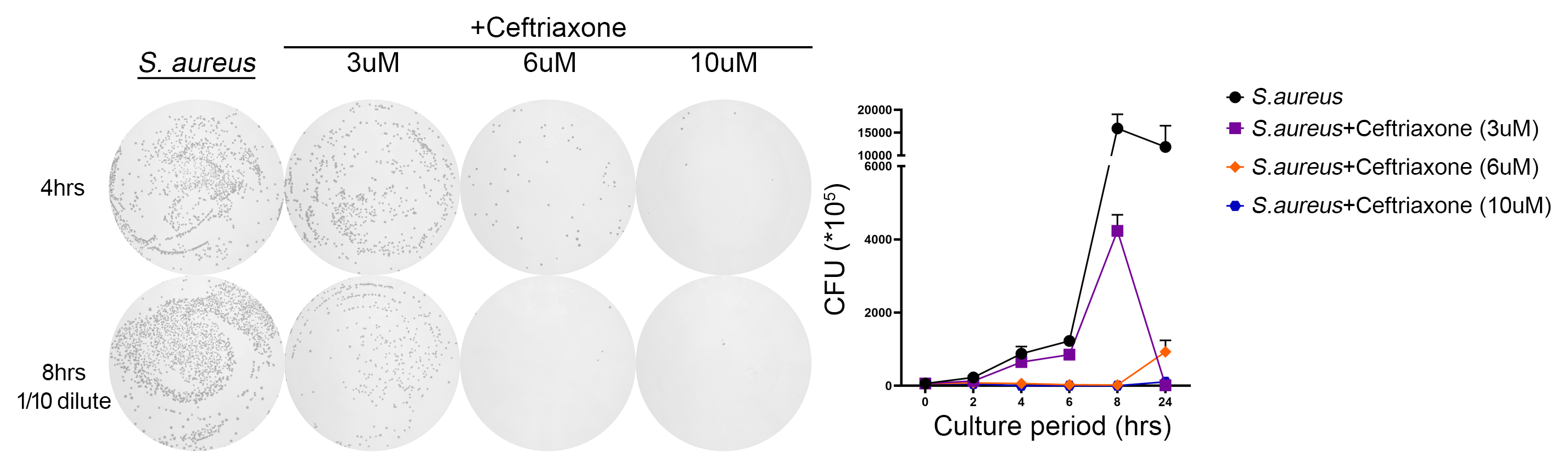

Supplement: szae034_suppl_Supplementary_Material [file szae034_suppl_supplementary_material.zip › Supplementary Figure/Supplementary Figure 1.tif]

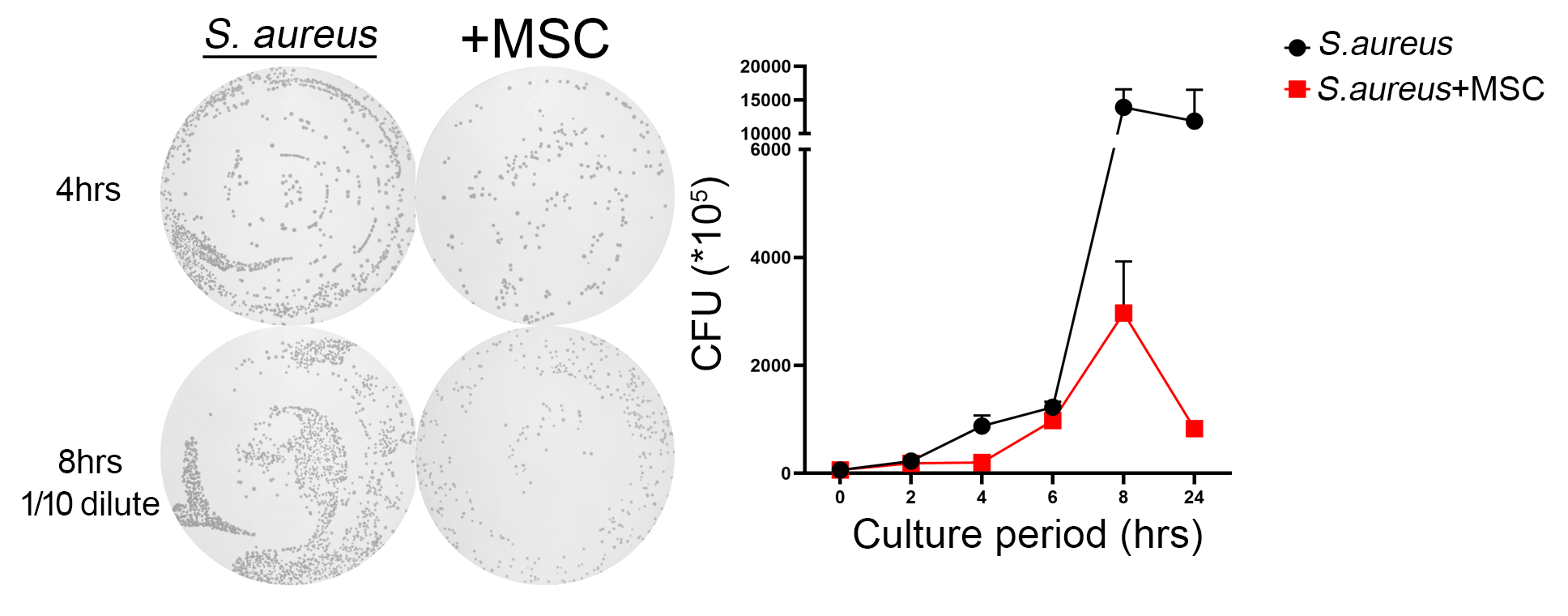

Supplement: szae034_suppl_Supplementary_Material [file szae034_suppl_supplementary_material.zip › Supplementary Figure/Supplementary Figure 2.tif]

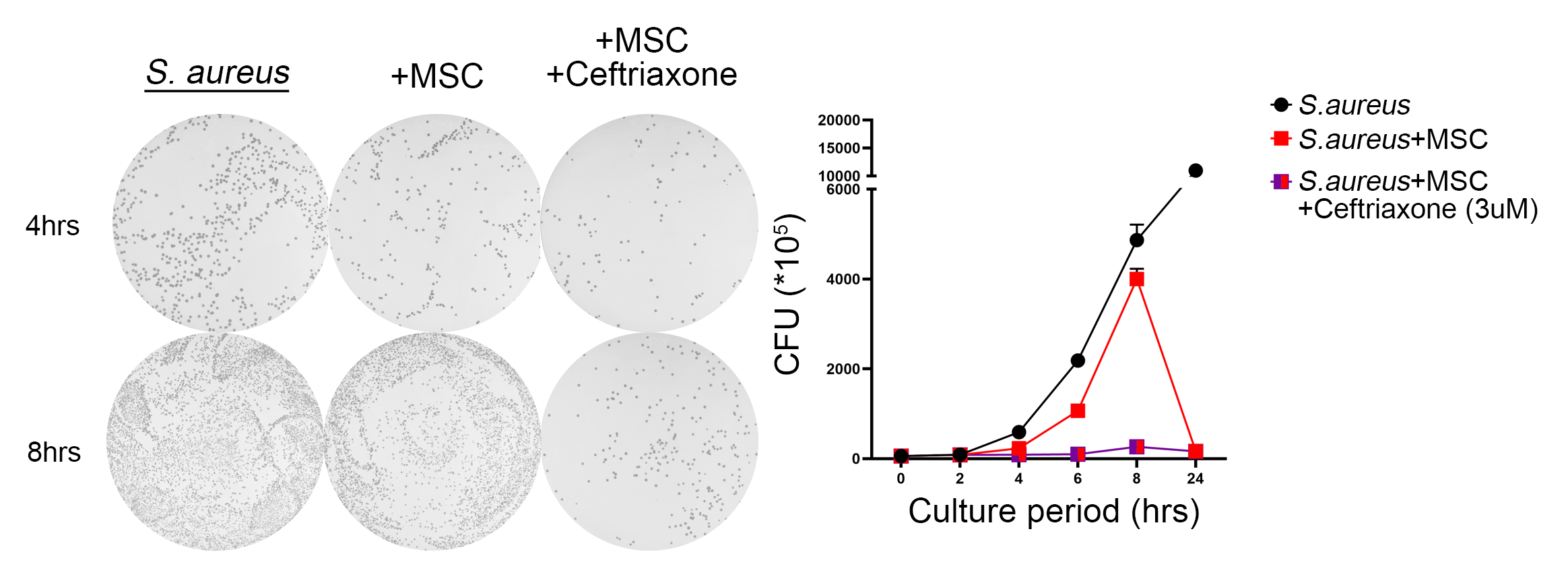

Supplement: szae034_suppl_Supplementary_Material [file szae034_suppl_supplementary_material.zip › Supplementary Figure/Supplementary Figure 3.tif]
